# Supplementary material for: A theory for self-sustained balanced states in absence of strong external currents
Source: PLoS Comput Biol. 2026 Feb 12;22(2):e1013465. doi: 10.1371/journal.pcbi.1013465 (PMC12923148; doi:10.1371/journal.pcbi.1013465)
Supplement: S1 Appendix — We show that different choices of transfer functions lead to the the same asymptotic values. (PDF) [file pcbi.1013465.s001.pdf]

S1 Appendix. Robustness of the results to the choice of the transfer function

In order to assess the robustness of the self-sustained balanced state with respect to the neuronal input–output relationship, we consider four different transfer functions commonly employed in rate-based and spiking network models. These transfer functions can be at zero input ($x = 0$) intrinsically active or strictly silent, however, with transfer functions that are always non negative.

1. Error function (erf). The transfer function used in the main text is defined as

$$\phi_{\mathrm{erf}}(x) = \frac{1}{2}\left(1 + \mathrm{erf}\left(\frac{x}{\sqrt{2}}\right)\right), \tag{S1-1}$$

This transfer function allows for a non-zero firing rate at zero input, reflecting intrinsic neuronal activity.

2. LIF-based transfer function. The second transfer function is derived from the steady-state firing rate of a leaky integrate-and-fire (LIF) neuron under constant input current, or equivalently when the synaptic dynamics is sufficiently slow, with respect to the membrane time constant.

$$\phi_{\mathrm{LIF}}(x) = \begin{cases} \left[-\tau_m \ln\left(\frac{x - v_{th}}{x - v_r}\right)\right]^{-1}, & x > v_{th}, \\ 0, & x \leq v_{th}, \end{cases} \tag{S1-2}$$

where τ_m is the membrane time constant, v_r the reset potential, and v_{th} the firing threshold. This transfer function satisfies the zero-input, zero-output condition.

3. Rectified Linear Unit (ReLU). As a simple prototypical threshold-linear transfer function, we consider the ReLU defined as

$$\phi_{\mathrm{ReLU}}(x) = \begin{cases} x, & x > 0, \\ 0, & x \leq 0, \end{cases} \tag{S1-3}$$

which enforces a strictly positive firing rate only for positive inputs and vanishes identically at zero input.

4. Quadratic transfer function. Finally, we consider a smooth nonlinear transfer function with a hard threshold,

$$\phi_{\mathrm{quad}}(x) = \begin{cases} x^2, & x > 0, \\ 0, & x \leq 0, \end{cases} \tag{S1-4}$$

which introduces a stronger nonlinearity while still satisfying the zero-input, zero-output property.

Asymptotic behavior

For each of the above transfer functions, we compute the steady-state firing rates of the excitatory and inhibitory populations, as well as the synaptic depression variable w, as functions of the network size N. As shown in Fig. S1-1, all transfer functions lead to the same asymptotic values in the thermodynamic limit $N \to \infty$. Differences across transfer functions are confined to finite-size effects and reflect distinct convergence paths toward the large-N regime, without affecting the existence or value of the self-sustained balanced state.

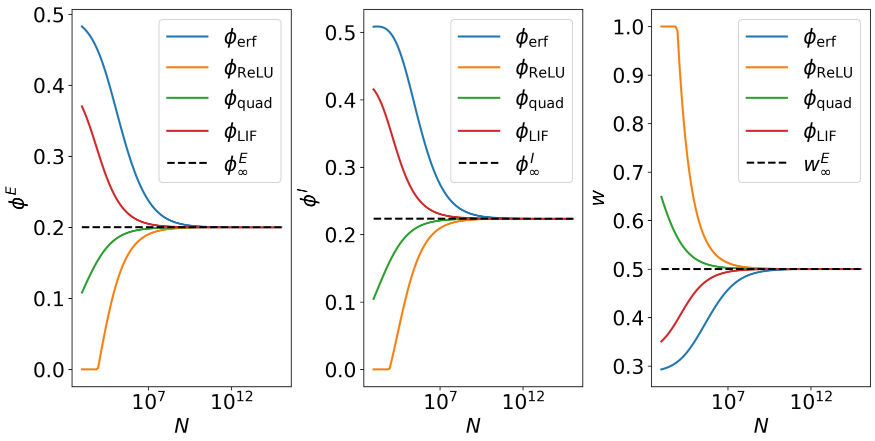

Figure S1-1: **Robustness of the self-sustained balanced state with respect to the neuronal transfer function.** Steady-state values as a function of network size N for four different transfer functions: error function (erf), LIF-based transfer function, ReLU, and quadratic transfer function. (A) Excitatory firing rate. (B) Inhibitory firing rate. (C) Synaptic depression variable w. In all panels, despite quantitative differences at finite sizes, the same thermodynamic-limit values are recovered as $N \to \infty$, demonstrating that the self-sustained balanced state does not depend on the specific choice of transfer function. In all panels $J_0 = 0.1$ and $I_0 = 0$, except for LIF transfer function where $I_0 = 1.1$ and where we set $\tau_m = 1$, $v_r = 0$ and $v_{th} = 1$. All other parameters as in the main text.
