# Supplementary material for: A theory for self-sustained balanced states in absence of strong external currents
Source: PLoS Comput Biol. 2026 Feb 12;22(2):e1013465. doi: 10.1371/journal.pcbi.1013465 (PMC12923148; doi:10.1371/journal.pcbi.1013465)
Supplement: S2 Appendix — We derive leading-order corrections to the asymptotic solutions by expanding the stationary population-averaged quantities in powers of ϵ=1/N, revealing how the external current I0 and synaptic coupling J0 influence the dynamics in large but finite networks. (PDF) [file pcbi.1013465.s002.pdf]

S2 Appendix. Finite-Size Corrections to the Asymptotic Balanced Solutions

In the main text, we have derived in the homogeneous stationary case the asymptotic solutions for the firing rates ($\phi_{\infty}^{E,I}$) and the synaptic depression variable (w_{∞}) in the thermodynamic limit $N \to \infty$ (Eqs. (6)). Here, we perform a perturbation analysis to quantify finite-size corrections to the asymptotic solutions. Specifically, we expand the population-averaged stationary solutions in powers of $\epsilon = 1/\sqrt{N}$ to determine how the external current I_0 and synaptic coupling J_0, not entering in the asymptotic solutions, influence the dynamics in large but finite networks without having to solve the set of implicit equations in (5).

We propose the following expansion to the first order in ε for the homogeneous stationary solutions:

$$\phi^E = \phi^{-1}(x^E) = \phi_E^{(0)} + \frac{1}{\sqrt{N}}\phi_E^{(1)} + \mathcal{O}\left(\frac{1}{N}\right), \tag{S2-1a}$$

$$\phi^I = \phi^{-1}(x^I) = \phi_I^{(0)} + \frac{1}{\sqrt{N}}\phi_I^{(1)} + \mathcal{O}\left(\frac{1}{N}\right), \tag{S2-1b}$$

$$w = w^{(0)} + \frac{1}{\sqrt{N}}w^{(1)} + \mathcal{O}\left(\frac{1}{N}\right), \tag{S2-1c}$$

where the zeroth-order terms correspond to the asymptotic solutions derived in the main text: $\phi_{E,I}^{(0)} \equiv \phi_{\infty}^{E,I}$ and $w^{(0)} \equiv w_{\infty}$. In LHS in (S2-1) we drop the 0 appearing in the main text to denote stationary solutions to avoid confusion.

Linearization of the Synaptic Depression The steady-state condition for the synaptic efficacy is given by Eq. (5c) :

$$w = \frac{1}{1 + \tau_D u \phi^E}. \tag{S2-2}$$

Linearizing around the asymptotic value $\phi_E^{(0)}$, we obtain the first-order correction for w:

$$w^{(1)} = \left.\frac{dw}{d\phi_E}\right|_{\phi_E^{(0)}} \phi_E^{(1)} = -\tau_D u \left(w^{(0)}\right)^2 \phi_E^{(1)}. \tag{S2-3}$$

Expansion of the Balanced Equations We substitute the expansions (S2-1a–S2-1c) into the mean-field stationary equations (5a) and (5b). Let $\mu_{\infty}^{E,I} \equiv x_{\infty}^{E,I} = \phi^{-1}[\phi_{E,I}^{(0)}]$ be the asymptotic input currents required to sustain the zeroth order firing rates. Rearranging Eq. (5a) and dividing by $\sqrt{N}$, we write:

$$\frac{\mu_{\infty}^E - I_0}{\sqrt{N}} = J_0 j_E \left(\sqrt{c_E}\phi^E w - g_E\sqrt{c_I}\phi^I\right). \tag{S2-4}$$

Substituting the perturbative ansatz and keeping terms up to order $\mathcal{O}(1/\sqrt{N})$, the LHS becomes $(\mu_\infty^E - I_0)/\sqrt{N}$. On the RHS, we expand the product $\phi^E w$:

$$\phi^E w \approx \phi_E^{(0)} w^{(0)} + \frac{1}{\sqrt{N}} \left(\phi_E^{(0)} w^{(1)} + w^{(0)} \phi_E^{(1)} \right). \tag{S2-5}$$

Using Eq. (S2-3) and the identity $w^{(0)} = 1 - \tau_D u \phi_E^{(0)} w^{(0)}$, the term in the parenthesis simplifies to $(w^{(0)})^2 \phi_E^{(1)}$.

By equating terms of order $1/\sqrt{N}$ for both the excitatory and inhibitory populations, we obtain the following linear system for the corrections $\phi_E^{(1)}$ and $\phi_I^{(1)}$:

$$\frac{\mu_\infty^E - I_0}{J_0 j_E} = \sqrt{c_E} (w^{(0)})^2 \phi_E^{(1)} - g_E \sqrt{c_I} \phi_I^{(1)}, \tag{S2-6a}$$

$$\frac{\mu_\infty^I - I_0}{J_0 j_I} = \sqrt{c_E} \phi_E^{(1)} - g_I \sqrt{c_I} \phi_I^{(1)}. \tag{S2-6b}$$

Solution for Finite-Size Corrections The system (S2-6a–S2-6b) can be written in matrix form $\mathbf{A}\vec{\phi}^{(1)} = \vec{b}$, with determinant $D = \sqrt{c_E c_I} g_E (1 - g_E/g_I)$. Solving for the first-order corrections yields:

$$\phi_E^{(1)} = \frac{1}{D} \left[\frac{g_I \sqrt{c_I}(I_0 - \mu_\infty^E)}{J_0 j_E} - \frac{g_E \sqrt{c_I}(I_0 - \mu_\infty^I)}{J_0 j_I} \right], \tag{S2-7a}$$

$$\phi_I^{(1)} = \frac{1}{D} \left[\frac{\sqrt{c_E}(I_0 - \mu_\infty^E)}{J_0 j_E} - \frac{\sqrt{c_E}(w^{(0)})^2 (I_0 - \mu_\infty^I)}{J_0 j_I} \right]. \tag{S2-7b}$$

These expressions explicitly demonstrate that for large, yet finite N, the first order corrections to the firing rates depend linearly on the external drive I_0 and are inversely proportional to the synaptic coupling J_0. This analytical result explains the finite-size trends observed in Fig. 2 of the main text. Figure S2-1 presents the comparison between the implicit self-consistent size-dependent fixed point solutions Eqs. (5), the asymptotic solution (zeroth-order) valid for $N \to \infty$ (Eqs. (6)) and the finite size correction (S2-1). As shown in this figure, the first order corrections in $\mathcal{O}(1/\sqrt{K})$ reproduce quite well the full implicit fixed point solutions (5) already for $N \simeq 10^7$, corresponding to $K_E \simeq 2.5 \times 10^5$ and $K_I \simeq 5 \times 10^4$

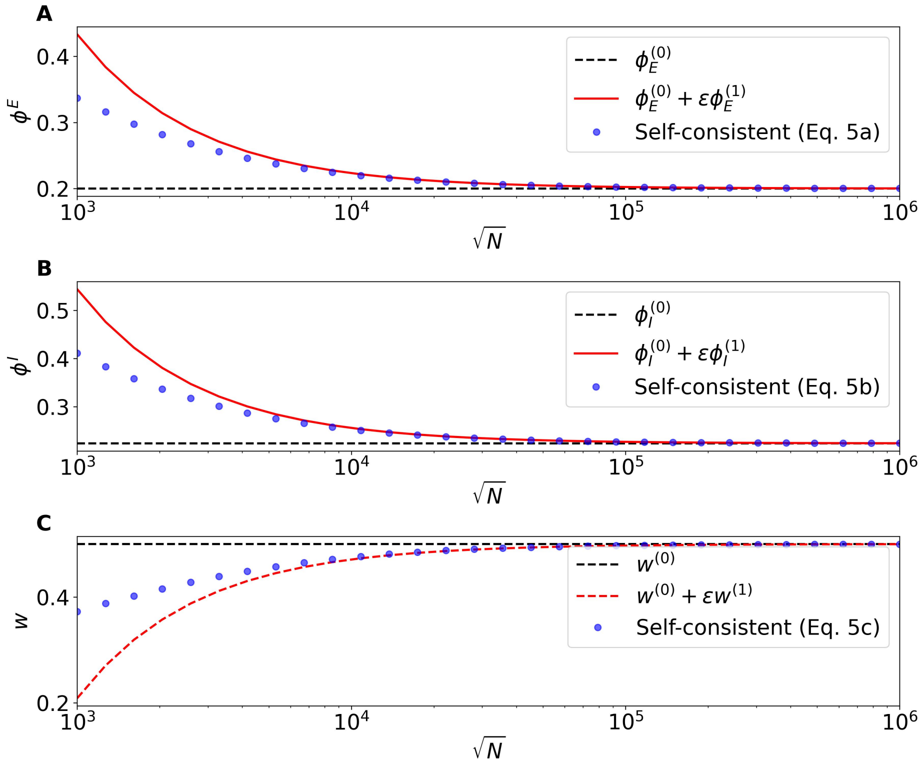

Figure S2-1: **Convergence to the thermodynamic limit and finite-size scaling.** Analytical and numerical comparison of the stationary solutions as a function of $\sqrt{N}$ $(1/\epsilon)$. The panels display (A) the excitatory firing rate ϕ^E, (B) the inhibitory firing rate ϕ^I, and (C) the synaptic depression variable w. Blue dots represent the exact self-consistent solution of the mean-field equations (5). The black dashed lines indicate the asymptotic zeroth-order prediction ($N \to \infty$, Eqs. (6)), while the red solid lines show the first-order perturbative approximation Eqs. (S2-1). The agreement between the red lines and the numerical data for small ϵ validates the $1/\sqrt{N}$ scaling of the finite-size corrections. Parameters: $J_0 = 0.1$, $I_0 = 0.5$, other parameters as in Table 1.
