# Supplementary material for: A theory for self-sustained balanced states in absence of strong external currents
Source: PLoS Comput Biol. 2026 Feb 12;22(2):e1013465. doi: 10.1371/journal.pcbi.1013465 (PMC12923148; doi:10.1371/journal.pcbi.1013465)
Supplement: S3 Appendix — We illustrate how the outlier eigenvalues depend on the system size N, comparing the predictions of the random matrix approximation with the eigenvalues obtained from direct diagonalization of the original Jacobian matrix DFhet. (PDF) [file pcbi.1013465.s003.pdf]

S3 Appendix. Outlier Eigenvalues for the Homogeneous Fixed Point

Figure S3-1 illustrates the dependence of the outlier eigenvalues on the system size N predicted by the random matrix approximation compared with those obtained from the diagonalization of the original Jacobian matrix $\boldsymbol{DF}_{\text{het}}$.

Panel A shows the leftmost outlier λ^{-}_{out} predicted by the random matrix approximation (orange dot), as given by Eq. (27) , alongside with the smallest eigenvalue of the full Jacobian λ_C^{min} (blue dot), which is expected to coincide with λ^{-}_{out} for the chosen parameters. Although there is a slight mismatch between the two curves, the 2 eigenvalues scale similarly with the system size. The inset displays the absolute value of both quantities on a log-log scale, revealing that they follow a power-law decay with N controlled by the same exponent $\gamma = 0.39$. This value is not too far from the theoretically expected scaling exponent of 0.5, due to the $\sqrt{N}$ factor present in the average matrix $\mathbf{M}$ in Eq. (26).

Panel B presents the behavior of the rightmost outlier λ^{+}_{out} obtained by the random matrix approximation (blue dot), compared with the largest eigenvalue of the original Jacobian, λ_C^{max} (orange dot). Unlike in panel A, there is no clear correspondence between λ^{+}_{out} and any specific eigenvalue of $\boldsymbol{DF}_{\text{het}}$, so we decided to track the largest eigenvalue as a proxy. The idea is that, if λ^{+}_{out} continues to grow with N, it might eventually become the the dominant eigenvalue for the instability of the homegeneous fixed point. However, as shown in panel B, this does not occur: λ_C^{max} indeed remains bounded for increasing N and does not follow the trend of λ^{+}_{out}, suggesting that the largest eigenvalue of the original system is indeed always contained within the bulk of the spectrum, regardless of N. This behavior points to the existence of a cancellation mechanism in the original dynamics that suppresses the emergence of the predicted outlier, likely missed by the zeroth-order approximation used in deriving the random matrix description.

Interestingly, although both λ^{-}_{out} and λ^{+}_{out} stem from the same average matrix $\mathbf{M}$ and could be expected to follow similar power-law scaling with N, λ^{+}_{out} appears to grow more slowly, as shown in the inset of panel B. There, a logarithmic fit provides a better description of its growth with the system size, suggesting the presence of additional nontrivial cancellation effects affecting the rightmost outlier, which, while not entirely suppressing its growth, do significantly slow it down.

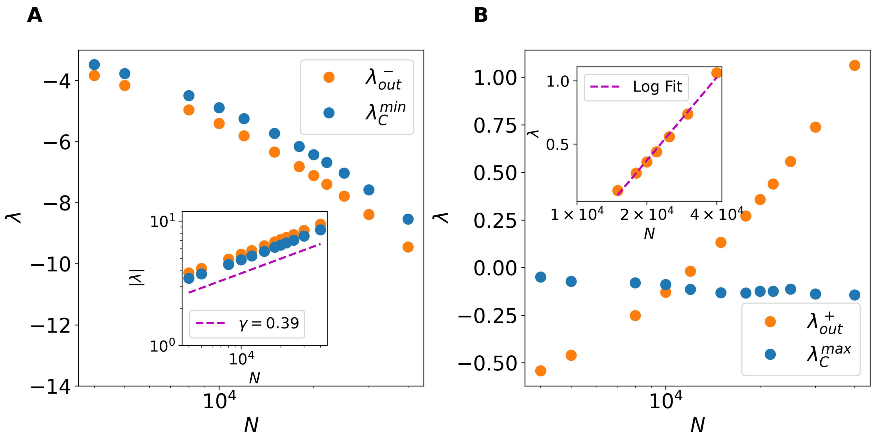

Figure S3-1: **Comparison between outlier predictions and direct diagonalization.** (A) Predicted leftmost outlier eigenvalue λ_{out}^{-} (blue) compared with the smallest eigenvalue obtained via direct diagonalization of the full Jacobian (orange), as a function of system size N. Inset: Absolute values of the same eigenvalues displayed in the main panel on a log-log scale, showing a power-law fit (offset for clarity) with exponent $\gamma = 0.39$. (B) Predicted rightmost outlier eigenvalue λ_{out}^{+} (blue) compared with the largest eigenvalue from direct diagonalization (orange), also as a function of N. Inset: λ_{out}^{+} restricted to its positive values, plotted on a semi-log scale, along with a logarithmic fit. In both panels, parameters are fixed at $J_0 = 0.8$ and $I_0 = 0$.
