# Supplementary material for: A theory for self-sustained balanced states in absence of strong external currents
Source: PLoS Comput Biol. 2026 Feb 12;22(2):e1013465. doi: 10.1371/journal.pcbi.1013465 (PMC12923148; doi:10.1371/journal.pcbi.1013465)
Supplement: S4 Appendix — We analyze the mismatch between the autocorrelation function (ACF) of the total inhibitory input predicted by Dynamical Mean-Field (DMF) theory and that obtained from direct network simulations. Our focus is on the role of finite network size and finite in-degree, considering sparsely connected networks with fixed numbers of excitatory (KE) and inhibitory (KI) pre-synaptic connections per neuron, while systematically varying the total network size N. (PDF) [file pcbi.1013465.s004.pdf]

S4 Appendix. Validity of the DMF approximation in finite size networks

In Figure S4-1, we analyze the mismatch between the autocorrelation function (ACF) for the total inhibitory input predicted by the Dynamic Mean-Field (DMF) theory and that obtained from direct network simulations, focusing on the impact of finite network size and a finite number of synaptic connections. We consider sparsely connected networks with a fixed number of pre-synaptic excitatory K_E and inhibitory K_I connections per neuron while systematically varying the total network size N.

Panel A shows how the ACF from direct simulations approaches the DMF prediction (black curves) as the network size increases. Different sub-panels illustrate this convergence for increasing values of K_E and K_I. The overall trend can be summarized as follows: networks with lower $K_{E,I}$ exhibit smaller discrepancies even at relatively small network sizes, while networks with higher connectivity require progressively larger N to match the DMF predictions closely. More sparse is the network better is the agreement, since the sparseness induces larger fluctuations in the input currents and the resulting dynamics of the neurons are less correlated and more stochastic, thus fulfilling better the hypothesis of the mean-field DMF theory.

Panel B summarizes these results by plotting the relative mismatch between the variance predicted by DMF theory and that obtained from direct simulations as a function of N for each connectivity scenario. The results confirm the expectation that using low values of $K_{E,I}$ (as adopted in the main text) favours accurate estimation of the ACF with relatively small network sizes. Notably, by considering a network with $N = 10,000$ and $K_I = 25$ and $K_E = 125$ the relative mismatch with respect to the DMF prediction can be already quite small (around 7%), demonstrating that accurate DMF approximations can be obtained with small, sparsely connected networks. This has important implications for computational feasibility, as highly sparse connectivity matrices enable efficient simulation using optimized ODE solvers while retaining quantitative agreement with DMF predictions.

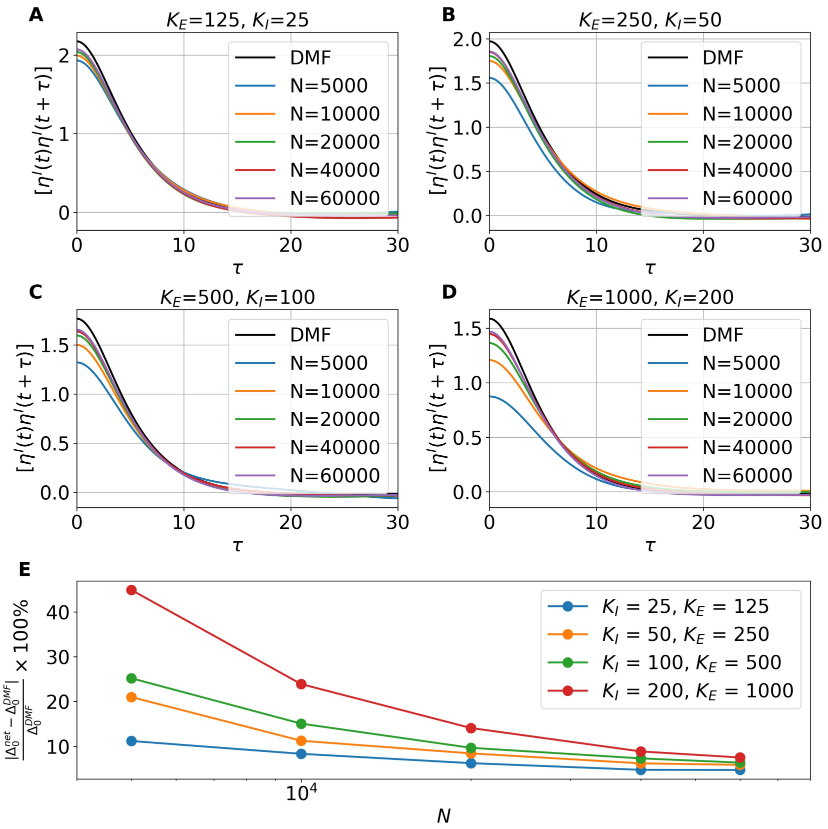

Figure S4-1: **Finite-size effects on DMF approximations.** (A-D) Population-averaged autocorrelation functions for the total inhibitory inputs from direct network simulations for increasing network sizes, compared with DMF predictions (black curves). Each subpanel corresponds to different values of the number of pre-synaptic excitatory and inhibitory connections per neuron, K_E and K_I, as indicated in the titles. (E) Relative percentage difference between the variance predicted by DMF approach and that obtained from direct simulations as a function of network size N, shown for the four connectivity scenarios reported in panels A-D. Direct network simulations were performed using $J_0 = 1.5$ and $I_0 = 0$, calculating the ACF over a time window of $t = 500$ after discarding a transient of 500 time units. Results were averaged over 8 independent network realizations for each N.
