# Supplementary material for: A theory for self-sustained balanced states in absence of strong external currents
Source: PLoS Comput Biol. 2026 Feb 12;22(2):e1013465. doi: 10.1371/journal.pcbi.1013465 (PMC12923148; doi:10.1371/journal.pcbi.1013465)
Supplement: S5 Appendix — We analyze how the width of the transition region depends on the network size using the largest Lyapunov Exponent. (PDF) [file pcbi.1013465.s005.pdf]

S5 Appendix. Generalizations of Synaptic Plasticity Rules: Compatibility with the Self-Sustained Balance

The analytical results demonstrating the existence of a non-trivial self-sustained balanced state (Eqs. (6)) rely on the specific architecture where Short-Term Depression (STD) modulates only the excitatory-to-excitatory synapses (J_{EE}). Here, we explore two generalizations of the plasticity rules. While we limit to analyze the homogeneous stable solution, we expect similar findings in the Rate Chaos regime.

Case 1: Asymmetric Plasticity affecting EE and II Synaptic Connections We first consider a system where STD is present on both excitatory-to-excitatory synapses (J_{EE}) and inhibitory-to-inhibitory synapses (J_{II}), while the cross-population connections (J_{EI} and J_{IE}) remain static. We introduce two distinct depression variables: w^E (for J_{EE}) and w^I (for J_{II}), each characterized by its own plasticity parameters $\tau_{D,\alpha}$ and u_α (for $\alpha \in \{E, I\}$).

Finite-Size Mean-Field Equations The stationary mean-field equations (generalizing Eq. (5) in the main text) are:

$$x_0^E = \sqrt{N} J_0 j_E \left(\sqrt{c_E}\phi[x_0^E] w_0^E - g_E \sqrt{c_I}\phi[x_0^I]\right) + I_0, \tag{S5-1}$$

$$x_0^I = \sqrt{N} J_0 j_I \left(\sqrt{c_E}\phi[x_0^E] - g_I \sqrt{c_I}\phi[x_0^I] w_0^I\right) + I_0. \tag{S5-2}$$

The steady-state synaptic availabilities are given by the presynaptic rates:

$$w_0^\alpha = \frac{1}{1 + \tau_{D,\alpha} u_\alpha \phi[x_0^\alpha]}, \quad \text{for } \alpha \in \{E, I\}. \tag{S5-3}$$

Asymptotic Solution in the Thermodynamic Limit In the limit $N \to \infty$, the balance conditions (by setting the $\mathcal{O}(\sqrt{N})$ terms to zero) are:

$$\sqrt{c_E}\phi_\infty^E w_\infty^E - g_E \sqrt{c_I}\phi_\infty^I = 0, \tag{S5-4}$$

$$\sqrt{c_E}\phi_\infty^E - g_I \sqrt{c_I}\phi_\infty^I w_\infty^I = 0. \tag{S5-5}$$

Substituting the inhibitory activity from the first equation into the second leads to the generalized equation of state for the synaptic efficacies:

$$w_\infty^E w_\infty^I = \frac{g_E}{g_I}. \tag{S5-6}$$

The self-sustained balanced state persists in this asymmetric configuration. The system adjusts the product of the synaptic availabilities to compensate for the gain mismatch g_E/g_I. The firing rates ϕ_∞^E and ϕ_∞^I are then uniquely determined by solving Eq. (S5-6) simultaneously with the definitions of $w_\infty^{E,I}$ as functions of $\phi_\infty^{E,I}$, which yields

$$\phi_\infty^E = \frac{1}{\tau_E u_E}\left(\frac{1}{w_\infty^E} - 1\right) \tag{S5-7}$$

$$\phi_\infty^I = \frac{1}{\tau_I u_I}\left(\frac{1}{w_\infty^I} - 1\right). \tag{S5-8}$$

Case 2: Symmetric Plasticity depending on the Nature of the Pre-synaptic Neurons We now consider a different scenario where the STD depends only on the pre-synaptic neuron type and therefore the same variables w^E and w^I modulate all the outgoing synapses. That is, w^E modulates J_{EE} and J_{IE}, while w^I modulates J_{EI} and J_{II}.

Finite-Size Mean-Field Equations The stationary mean-field equations become:

$$x_0^E = \sqrt{N} J_0 j_E \left(\sqrt{c_E}\phi[x_0^E] w_0^E - g_E\sqrt{c_I}\phi[x_0^I] w_0^I\right) + I_0, \tag{S5-9}$$

$$x_0^I = \sqrt{N} J_0 j_I \left(\sqrt{c_E}\phi[x_0^E] w_0^E - g_I\sqrt{c_I}\phi[x_0^I] w_0^I\right) + I_0. \tag{S5-10}$$

Asymptotic Solution and Loss of Balance In the thermodynamic limit, the balance conditions are:

$$\sqrt{c_E}\phi_\infty^E w_\infty^E = g_E\sqrt{c_I}\phi_\infty^I w_\infty^I, \tag{S5-11}$$

$$\sqrt{c_E}\phi_\infty^E w_\infty^E = g_I\sqrt{c_I}\phi_\infty^I w_\infty^I. \tag{S5-12}$$

Equating the right-hand sides of both equations yields:

$$g_E\sqrt{c_I}\phi_\infty^I w_\infty^I = g_I\sqrt{c_I}\phi_\infty^I w_\infty^I. \tag{S5-13}$$

This simplifies to:

$$(g_I - g_E)\sqrt{c_I}\phi_\infty^I w_\infty^I = 0. \tag{S5-14}$$

Since we assume the necessary condition for balanced networks $g_I \neq g_E$ (gain-mismatch) and that $\sqrt{c_I} > 0$ and $w_\infty^I > 0$ (plasticity not zero), the only solution that satisfies the balance condition is the trivial quiescent state:

$$\phi_\infty^I = 0 \quad \text{and consequently} \quad \phi_\infty^E = 0. \tag{S5-15}$$

This demonstrates that the self-sustained balanced state breaks down under this symmetric plasticity scheme. The existence of the non-trivial state requires an asymmetry in how the depression variables modulate the excitatory (inhibitory) currents received by the excitatory and inhibitory populations. This can be obtained by assuming e.g. different time scales for the evolution of the STD associated to EE and IE connections, as well as for those associated to II and EI ones.

In view of the differential depression reported for excitatory and inhibitory synapses onto visual cortex pyramidal neurons [1], it will be particular interesting for future studies to examine EE and EI connections modulated by STD with different synaptic time scales and their influence on the balanced dynamics.

References

[1] Varela JA, Song S, Turrigiano GG, Nelson SB. Differential depression at excitatory and inhibitory synapses in visual cortex. Journal of Neuroscience. 1999;19(11):4293–4304.
