# Supplementary material for: A theory for self-sustained balanced states in absence of strong external currents
Source: PLoS Comput Biol. 2026 Feb 12;22(2):e1013465. doi: 10.1371/journal.pcbi.1013465 (PMC12923148; doi:10.1371/journal.pcbi.1013465)
Supplement: S6 Appendix — The analytical results demonstrating the existence of a non-trivial self-sustained balanced state (Eq (6)) rely on a specific architecture where Short-Term Depression (STD) acts only on excitatory-to-excitatory synapses (JEE). Here, we explore two generalizations of the synaptic plasticity rules. (PDF) [file pcbi.1013465.s006.pdf]

S6 Appendix. Dependence of the Width of the Transition Region on the Network Size

To analyze how the width of the transition region depends on the network size, we computed the median largest Lyapunov exponent, $\tilde{\Lambda}_1$, across 20 network realizations as a function of the synaptic coupling strength J_0. This indicator allows us to accurately estimate the synaptic coupling values associated with the loss of stability of the homogeneous fixed point, J_c, and the onset of *rate chaos*, which we denote as J_r. Figure S6-1A shows $\tilde{\Lambda}_1$ for three representative network sizes. The first transition, indicated by J_c, corresponds to the point where the median $\tilde{\Lambda}_1$ crosses from negative values to near zero, in agreement with the theoretical prediction for the loss of stability of the homogenous fixed point given by Eq. (30). The second transition, J_r, is operationally defined as the minimal value of J_0 for which $\tilde{\Lambda}_1$ remains strictly positive for any $J_0 > J_r$, signaling the emergence of a robust chaotic phase.

Furthermore, as shown in Fig. S6-1B J_c and J_r appraoch each other for increasing N. As evident, from the inset of panel B, the width of the transition region $J_r - J_c$ shrinks for increasing system sizes. Thus suggesting that in the thermodynamic limit one could eventually observe an abrut transition from a stable fixed point solution to a chaotic regime.

It is worth noticing that we do not expect the trend of J_r to be strictly monotonically decreasing, since—as shown in Fig. 2—J_c continues to increase beyond 1 as $N \to \infty$. Consequently, we believe that J_r will eventually modify its evolution to satisfy $J_r > J_c$, while the width of the transition region will continue to shrink.

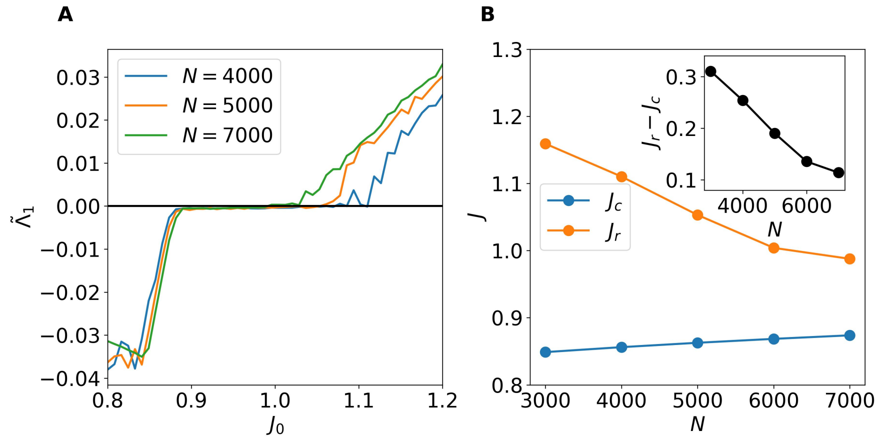

Figure S6-1: **Network size influence on the width of the transition region.** (A) Median largest Lyapunov exponent $\tilde{\Lambda}_1$ as a function of synaptic coupling J_0 for three representative network sizes. (B) Evolution of J_c and J_r with the network size N. Inset: Width of the transition region $J_r - J_c$ as a function of N. For these results, $\tilde{\Lambda}_1$ was computed over 20 different network realizations for each N, with Lyapunov exponents evaluated over 60,000 time units following a transient of 300 time units to ensure convergence.
