# Supplementary material for: A theory for self-sustained balanced states in absence of strong external currents
Source: PLoS Comput Biol. 2026 Feb 12;22(2):e1013465. doi: 10.1371/journal.pcbi.1013465 (PMC12923148; doi:10.1371/journal.pcbi.1013465)
Supplement: Attachment — Submitted filename: Referee_Reply.pdf [file pcbi.1013465.s007.pdf]

# Detailed Replies to All the Reviewers' Questions

**Reviewer #1**: In this manuscript, the authors consider networks of excitatory and inhibitory neurons in a balanced state at large system size. Unlike previous work in which external inputs to the network is strong in the limit of large network size, their model produces self-sustained non-vanishing activity in the large network limit with weak or absent external input. This is achieved with short term synaptic depression on synapses between excitatory neurons. The authors use asymptotic analysis and computer simulations to explore steady-state and non-steady state dynamics, for example chaos, in the networks.

The manuscript makes a novel contribution to the mathematical analysis of these types of networks.

**Major comments:**

A central motivation for the models studied in this manuscript is that traditional balanced networks require that mean external input to each neuron scales like O(sqrt(K)) where K is the in-degree of a typical neuron. The authors are correct to point out that this has been challenged by recent theoretical work (refs 9 and 10) and that it might contradict some experimental evidence (refs 11-15), but their discussion of these points lacks nuance in such a way that it could mislead readers who are not familiar with the literature on balanced networks.

(Q1) First, the authors fail to acknowledge that strong external input follows from modeling feed-forward synaptic connections in the same way that they model recurrent connections: If neurons receive O(sqrt K) feed-forward synaptic inputs and feed-forward synaptic weights scale like 1/sqrt(K), like they have assumed of recurrent connectivity, then O(sqrt K) external input follows. If feed-forward input is O(1) then feed-forward synaptic inputs must either be sparser (O(1) in number instead of O(K)) or weaker (O(1/K) in strength instead of O(1/sqrt K)) than recurrent synaptic inputs. The authors should acknowledge this point and cite any evidence they have for one or the other possibility (sparser or weaker feed-forward input).

**(R1) The Reviewer is right, we have now explicitly acknowledged this aspect in the Introduction. Furthermore, in the Discussion we have suggested that for the reported experiments the feed-forward inputs could be indeed sparser then in the cortex, namely we wrote :**

***As already mentioned in the Introduction, strong external currents follows from assuming for the feed-forward connections an out-degree O(K) and synaptic strengths O(1/√K). If the external current is weak, one of this two hypotheses should be not true. Most of the experimental evidences showing that input currents are O(1) involve thalamo-cortical connections. In particular, it has been shown that the thalamic net excitation to the mice visual cortex, with optogenetic silencing of intra-cortical excitatory input, induce a depolarization that is of the order of the typical distance to threshold [9,13,14]. This implies that the feed-forward input is O(1) [10]. Evidences that the out-degree from thalamus to cortex is smaller than in the cortico-thalamo-cortical loop have been reported for macaque in [40]. Quite recently the number of thalamic neurons converging to a cortical neuron in the primary visual cortex of mouse has been***

***estimated giving out-degrees ranging from to 2-7 and accounting for the 90% of the total synaptic weight to the cortical neuron [41]. This result should be compared with a previous study indicating a mean out-degree of $\simeq$80 for thalamic inputs into layer 4 neurons [42]. Apart the exact value of the out-degree that can be affected by the method employed to estimate it (for a detailed discussion see [41]) it is clear that the feed-forward thalamo-cortical connections are definitely sparser than in the cortex. This seems to suggest that the hypothesis that the out-degree is O(K) is not valid, at least in this specific case, and this could be at the origin of the measured O(1) external currents***

(Q2) On a similar note, the authors' model predicts that the average recurrent input from excitatory neurons is large (and similarly for inhibitory neurons), so feedforward input is vanishingly weak compared to each of these two sources of recurrent input. Specifically, their model assumes that recurrent excitation and recurrent inhibition are each O(sqrt K). While this fact does follow from their equations, it is never mentioned, or at least it is not mentioned where the relative strengths of feedforward and recurrent input is discussed (eg, in the second paragraph of the Introduction). The prediction that feedforward input is vanishingly small compared to recurrent input is a fundamental prediction of their model, and it should be mentioned explicitly (for example, in the second paragraph of the Introduction, after eq 5, and/or in the Discussion section).

**(R2) We thank the Reviewer for this important observation. We agree that this should be stated explicitly in the main text, as it is a central prediction of the model.**

**We have now clarified this point in several places throughout the manuscript:**

- **In the second paragraph of the *Introduction*, where the scaling of feedforward vs. recurrent input is first discussed;**
- **Below Eq. (5), where the scaling naturally emerges from the expressions for the mean input currents;**
- **In the description of Figure 2C, where we illustrate the balance of excitatory and inhibitory inputs;**
- **And finally, in the *Discussion* section, where we reflect on the biological implications of this scaling behavior.**

(Q3) In Eq 6, it is shown that steady state rates in the limit do not depend on the stimulus, I0. This would, of course, be an undesirable property of a neural network since the network should respond to stimuli. The authors later show in Fig 2 that the network does respond to the stimulus whenever N is finite and smaller in scale than one trillion (10^12). As it turns out, the asymptotic steady states in Eq 6 are actually quite inaccurate for reasonable parameter regimes (when N=10^12, neurons receive literally billions of synaptic inputs, which around a million times larger than reality). I think this point should be acknowledged. Specifically, the N=10^12 results are perhaps interesting from a mathematical perspective to verify the asymptotic equations, but the realistic values of N are much smaller, and do not adhere to the asymptotic steady state.

**(R3) We thank the Reviewer for pointing this out. We have now stressed that realistic values of the in-degree would be definitely smaller. However, this is true for the stationary homogeneous solution, which are not particularly realistic. For the chaotic solution , the**

**convergence to the asymptotic balanced regime occurs already around N=10^6, as shown in Fig 7 A, where we employed DMF theory to solve the equations. And for N=10^6 for our dilution we get K_E and K_I of order 10^3 – 10^4 somehow in agreement with experimental indications. Furthermore, the self-consistent stationary solution (5) depends on the system size, on I0 and J0, and it can be obtained numerically and this is the solid line in Fig 2 A,C, D and the agreement with numerical solutions of the equations (solid circles) is perfect up to N=120,000 as shown in Fig 2 A.**

**Finally, as detailed in the next answer we have developed a finite size expansion in 1/sqrt(N) of the stationary solution reported in (5) to obtain analytic equations -not implicit ones as in (5)- that do depend on J0 and I0.**

(Q4) On a similar note to the previous comment, I0 does not appear in Eq 6 because it is omitted from the sqrt K scaling in Eq 5. However, a more accurate approximation could be obtaine by moving the the terms I0/(sqrt(N)*J0*jX) into the parenthesis in Eq 5 and then solving the resulting linear equation to produce a version of Eq 6 that depends on N (the equation for w0 might need to be derived differently). This approximation could be plotted in Fig 2 panels C and D alongside the existing approximations. It would be worth checking whether this gives a more accurate approximation than Eq 5, particularly at larger values of I0.

**(R4) We thank the Reviewer for suggesting this improvement. We have now added a new Appendix (now Appendix 1) devoted to the analysis of the first order corrections (in 1/sqrt(N)) to the asymptotic balanced results. Indeed, as expected by the Referee, this correction gives rates that are proportional to I0 and inversely proportional to J0, as we have observed with the exact self-consistent solutions reported in Eq. 5 and reported in Fig. 2. In particular, in Fig. 12 we have compared this 1st order approximation to the exact self consistent solution of Eq. (5) (reported also in Fig. 2) . From Fig. 12, it is evident that the 1st order correction agrees with the exact mean field results already at N of order of N=10^7, i.e. K_e = 2.5*10^5 and K_i = 5*10^4.**

(Q5) On a related note, how does the magnitude of I0 compare to the magnitude of the total input in Fig 2 C and D? The authors' motivation for their model is that I0 is comparable to the total input (and therefore much smaller than the recurrent excitatory and recurrent inhibitory inputs individually). This is clearly accurate when I0=0 and when N=10^12, but how accurate is it for more reasonable values of N when I0 is larger? The authors could plot this ratio (either I0 versus total, or I0 versus recurrent exc) for the simulations in Fig 2C and D, either as a supplementary figure or within the main text.

**(R5) By following the Reviewer’s suggestion we added a new inset in Fig 2C where we report the value of the (stationary) excitatory input current \mu^E versus I_0 for different system sizes, we observe that they are both of the same order for all considered sizes N. Furthermore, the input excitatory and inhibitory currents \mu^{E,I} are reported in Fig. 7 E in the chaotic regime, versus J0 for different values of I_0 at N=5000. Also in this case the input currents have values of the order of the unity comparable with the external drives. Finally, in Fig 8D are reported for the rate chaos the mean excitatory and inhibitory currents divided by sqrt(N) as a function of the system size, showing that they decay indeed as 1/sqrt(N). Thus indicating that the total input currents \mu^{E,I} achieve a constant value in the thermodynamic limit and they do not diverge or vanish.**

**Minor comments:**

(Q6) In the abstract, when the authors write "this novel balancing mechanism", it reads as if they are referring to rate chaos, whereas I am sure that they mean to refer to synaptic depression. It could be rephrase for clarity.

**(R6) We thank the Reviewer to point this out. Indeed, we have rephrased several parts of the abstract to make it clearer.**

(Q7) The authors consider EE short term plasticity, but not EI, IE, or II. Can the authors comment on this? Would other forms of short term plasticity achieve similar results? What if they were all combined into one model?

**(R7) By following the Reviewer's suggestions we have analyzed the asymptotic response of the network in 2 different scenarios in Appendix 5. In the first scenario STD is acting on EE and II connections, with STDs characterized by different parameters, also in this case we have found that the balanced state can exist and the conditions for its existence have been derived. In the second scenario we have considered the case where STD is present in all connections (EE,EI,IE and II) and where the STD characteristics are controlled only by the nature of the pre-synaptic neurons. Therefore the same "excitatory" STD will control EE and EI synapses, while an "inhibitory" STD will control the IE and II synapses. In this latter case the balanced state with a finite firing rate is not sustainable, and to be achieved we require to break the symmetry of the excitatory (inhibitory) driven STD. Therefore we expect that also for 4 different types of STD, one for each type of synapse, the balanced state can be finally achieved, but we let this analysis to future work.**

(Q8) The Discussion section appears after the Methods section. This seems unusual and interrupts the reading process.

**(R8) We thank the Reviewer for pointing this out, it was indeed a mistake, we have corrected for this.**

**Reviewer #2:** The article examines the dynamics that arise from incorporating short-term synaptic depression (STD) between excitatory–excitatory (E–E) neuron pairs in a neural rate-based network. The authors analyze the homogeneous and heterogeneous fixed points, as well as the perturbations around them. They also study the transition to chaos in finite-size networks and the convergence of this transition in the limit of large (effectively infinite) network size.

Overall, the manuscript is mathematically sound, and the derivations are clear and consistent. However, the writing and logical flow are often difficult to follow.

(Q1) My main concern is that it is difficult to identify the contribution of this study. The finite-size effects discussed here are well known for rate models and can arise even without including STD. The mean-field treatment and the use of eigenvalue spectra are also standard tools in this literature. As written, it is not evident what new insight is provided specifically by the inclusion of STD. The term “massively connected” corresponds to all-to-all connectivity, although it is well established that sparse random connectivity with the same weight statistics yields identical dynamics (up to scaling by sparsity). Most of (and possibly all) the reported phenomena appear qualitatively reproducible with simpler rate models lacking STD.

**(R1) The main scope of our paper it to show that a self-sustained balanced state can emerge also in absence of strong external currents, this is a quite timely subject since 2 recent papers by renown scientists investigate exactly this point (see [8] and [9]). Our contribution is that balancing can be achieved (and sustained) also in absence of strong external currents thanks to some biologically plausible *nonlinear* mechanism involving the neuronal firing rates. We have proposed here STD on excitatory-excitatory connections as a plausible mechanism, but other biological mechanism (e.g. frequency adaptation) can lead to the same result. Indeed as we have shown in Appendix 5, the balancing can be achieved also with two distinct depression mechanism acting on excitatory and inhibitory neurons. The quite surprising result is that despite the mechanism based on STD is completely different from the canonical one examined in [4,5,7] and based on strong external and strong recurrent excitatory and inhibitory currents, the observed dynamical scenario resembles the ones reported in [4] and specifically in [5] , where massively coupled networks have been investigated. Indeed most of the reported phenomena are qualitatively similar to those obtained with rate models in absence of STD, but these rate models require strong external currents growing as sqrt(K) to achieve a balanced dynamics, this is exactly the point. To our knowledge, this is the first work to analyze the full dynamical transitions from fixed point to chaos of this self-sustained balanced regime based on a nonlinear mechanism, namely on STD.**

**From a technical point of view we have extended Girko’s Law (employed to analyze the stability of stationary solutions) and Dynamical Mean Field (DMF) theory (used to characterize rate chaos) to excitatory-inhibitory networks with STD. These generalizations are to our knowledge new and not reported in literature previously.**

**Furthermore, we have performed a finite-size scaling of the transition to chaos via Lyapunov analysis: We have analyzed, for the first time to our knowledge, how the *transition region* between stable and chaotic regimes shrinks with increasing system size. This is achieved through systematic computation of the maximal Lyapunov exponent.**

**For what concerns "massively connected networks", these have been examined in the context of the classical balance theory in [7]: Renart A, De La Rocha J, Bartho P, Hollender L, Parga N, Reyes A, et al. *The asynchronous state in cortical circuits.* Science. 2010;327(5965):587–590. In this paper , the author employed the term "densely connected networks" instead of "massively coupled networks", but the framework is the same. They show that the balancing mechanism act in a completely different way with respect to that usually observed in sparse networks in [4,5]. In particular, while in asynchronous networks the cancellation of the large excitatory and inhibitory population currents occurs on average, in densely connected networks this occurs via an "almost instantaneous" tracking of spontaneous fluctuations in the activity of excitatory and inhibitory populations currents which cancel the effect of a large amount of shared inputs. In our model with STD we observe a similar scenario, despite the absence of strong external drives present in [7], a scenario reported also in experimental investigations of in vitro neural cultures (see [8]). To avoid misunderstanding we have substituted everywhere in the text *massively coupled networks* with *densely connected recurrent networks.***

(Q2) For revision, I suggest that the authors clearly delineate the novel contributions of the work. In particular, they should emphasize what genuinely new dynamical phenomena arise in the large-N limit due to STD, and why these cannot be obtained from standard rate models.

**(R2) By following the request of Reviewer 1 and 2 we have rewritten Abstract, Introduction and Summary, as well as added two new Appendices and new figures to render our message clearer. We hope that the manuscript is now more readable and clear.**
