# Supplementary material for: A theory for self-sustained balanced states in absence of strong external currents
Source: PLoS Comput Biol. 2026 Feb 12;22(2):e1013465. doi: 10.1371/journal.pcbi.1013465 (PMC12923148; doi:10.1371/journal.pcbi.1013465)
Supplement: Attachment — Submitted filename: Reply_Letter.pdf [file pcbi.1013465.s008.pdf]

Response to the Editor and Reviewers

Dear Editor,

The authors are grateful for the positive evaluation of our manuscript by both reviewers. In particular:

- **Reviewer #1** stated: *"The authors have addressed all of my comments."*
- **Reviewer #2** expressed that she/he is now *"convinced of the interest"* of the work.

We also note with appreciation that Dr. Robert Rosenbaum (Reviewer #1), a recognized expert in the theory of dynamically balanced networks, expressed satisfaction with the modifications introduced in the revised manuscript.

At the same time, we acknowledge the Editor's observation that *"there are some places where the manuscript needs work both in the presentation of the results and claims that have been made."* and also the additional Reviewer's comment on the organization of the manuscript. We have carefully addressed all the remarks point by point, as detailed below.

We are therefore resubmitting the manuscript, modified by following the Editor and Reviewer request, for publication in PLOS Computational Biology

Sincerely,
David Angulo Garcia and Alessandro Torcini

Detailed Replies to all the Questions/Comments

Reviewer's Comments

Q1: The reviewer # 2 suggests that the manuscript should first present results in the limit of large N ("infinite" solution), and only later discuss finite-size effects. While finite-size effects are interesting, they are not unique to this model.

R1: We have tried to follow the reviewer's suggestion regarding the organization of the manuscript and its clarity. To this end we have reconsidered the relative placement of the two regimes that persist in the infinite size limit - (i.e. the stable homogeneous fixed point solution and the rate chaos) with respect to the transition region that vanishes in the thermodynamic limit. Specifically, we have moved the sub-section describing the transition from the homogeneous fixed point to the rate chaos regime to a later position in the manuscript, placing it right before the sub-section devoted to the spiking neural network model (see the modified structure highlighted in red in the revised version). We have decided to leave the Spiking Neural Network as the final sub-section of the results as we believe it represents an evidence that the regimes found in rate models can be found also in a more realistic set-up, represented by spiking neural neworks.

We would like to clarify that the results shown in the first two sub-sections (referring to homogeneous stable fixed point and rate chaos) still discuss the dependence of the solutions on finite N, because the scaling of the solutions with the system size is a fundamental aspect of how the balanced regimes emerge.

We hope that this revised ordering harmonizes the reviewer's concern about organization and legibility with the authors' creative decision of building a coherent narrative which is the result of a deliberate effort carried out over more than three years. This organization reflects a narrative strategy that has been shaped through extensive review of relevant literature in the field (see Refs. [4-10], [20-21],[23-28],[30] and [34]) and is intended to present a comprehensive and logically structured account of the results. We fully agree that alternative ways of organizing the manuscript may also be reasonable and scientifically valid. However, we believe that a research paper is also a creative endeavor, and the choice of structure and narrative ultimately remains a decision of the authors, as long as the choice reflects scientific rigor and correctness -which both reviewers and the editor acknowledge-. For these reasons, we respectfully yet firmly believe that the current organization should be appropriate and well aligned with the scope and depth of the study

Editor Comments

Q1: The main claim of the manuscript is the emergence of self-sustained activity, which by definition would mean $I_0 = 0$. Then authors argue that a balanced state emerges for weaker feedforward inputs, unlike more classical work on this topic. While the mathematical analysis is correct, authors should note that the neuron transfer function (eq4) allows the neuron to have a nonzero response for zero input. So the claims about self-sustained activity and balanced state for weak inputs are a direct consequence of the neuron's transfer function choice. If neurons are intrinsically active (eq4) then self-sustained activity can arise even without connectivity. If the authors want to make the two aforementioned claims they must show the results for a realistic neuron transfer function (zero firing rate for zero input and strictly positive firing rate). And also please show the self-sustained activity for LIF neuron network.

R1: The Editor is correct in noting that, by definition, self-sustained activity corresponds to the case $I_0 = 0$ or at very least $\mathcal{O}(1)$ in a network with very large number of connections $K \to \infty$. Our claim is made precisely in this sense, and we maintain it based on the following arguments: While it is true that the specific form of the neuronal transfer function in Eq. (4) allows for a non-zero firing rate at zero input, this choice is not responsible for the emergence of the self-sustained balanced state in the thermodynamic limit. Indeed, Eqs. (6a–6c) explicitly show that the asymptotic firing rates are independent of the detailed shape of the transfer function. Different transfer functions may affect the manner in which the system approaches the asymptotic state for finite N, but not the value of the asymptotic firing rate itself (see Figure 1 in this letter).

To further substantiate this point, we have added Supplementary Information S1, where we report the steady-state firing rates as a function of network size N for four different transfer functions: the error function, LIF-based transfer function, a ReLU, a quadratic transfer function -notice that all of them except for the erf have the property of zero-input, zero-output and that they all are non negative. These results demonstrate that, despite quantitative differences at finite sizes, all the considered cases converge to the same thermodynamic-limit behavior.

Regarding the specific case of the LIF network, an important clarification is in order. In this case we need the requirement of a nonzero I_0 exceeding the LIF threshold since the threshold acts as an effective baseline that enables spike generation. We notice that the emergence of sustained activity remains a collective, network-driven phenomenon which is achieved for $\mathcal{O}(1)$ even for $K \to \infty$. We hope that this clarification adequately addresses the Editor's concerns and makes clear that our

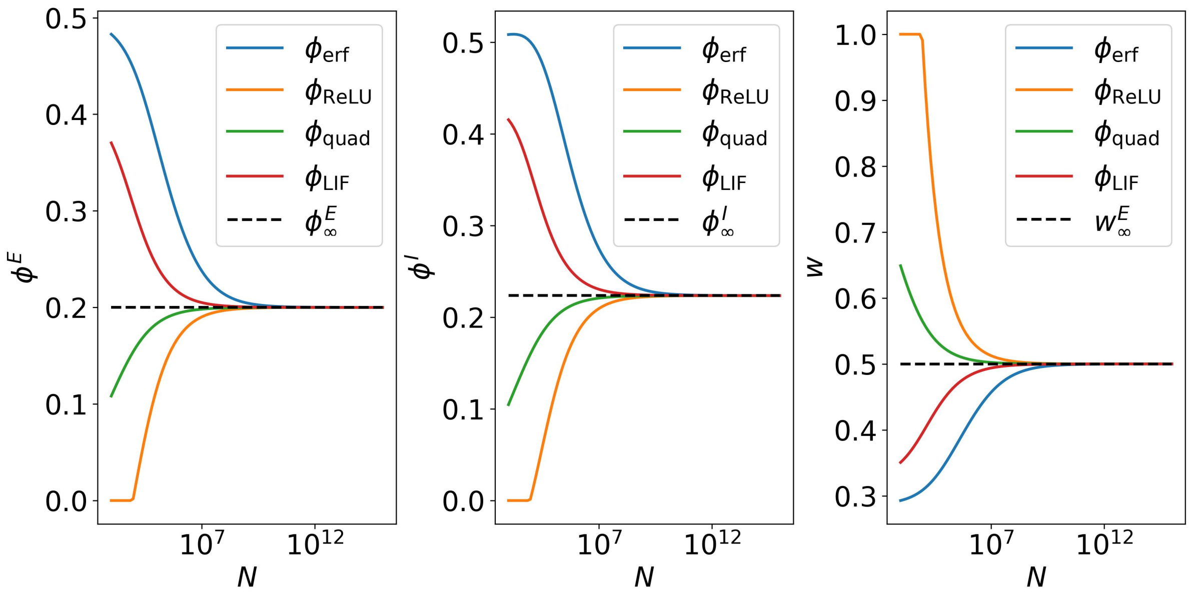

Figure 1: **Robustness of the self-sustained balanced state with respect to the neuronal transfer function.** Steady-state values as a function of network size N for four different transfer functions: error function (erf), LIF-based transfer function, ReLU, and quadratic transfer function. (A) Excitatory firing rate. (B) Inhibitory firing rate. (C) Synaptic depression variable w. In all panels, despite quantitative differences at finite sizes, the same thermodynamic-limit values are recovered as $N \to \infty$, demonstrating that the self-sustained balanced state does not depend on the specific choice of transfer function. In all panels $J_0 = 0.1$ and $I_0 = 0$, except for LIF transfer function where $I_0 = 1.1$ and where we set $\tau_m = 1$, $v_r = 0$ and $v_{th} = 1$. All other parameters as in the main text.

conclusions do not hinge on a particular choice of neuronal transfer function. We have made this explicit in the manuscript in the following sentences:

The balanced steady-state firing rates do not depend on the specific form of the transfer function, but only on the coupling parameters (g_E, g_I), the connectivity densities (c_E, c_I), and the STD parameters. As shown in Appendix S1, the same thermodynamic-limit solutions are obtained for different classes of transfer functions; the only difference among them lies in the manner in which the large-N limit is approached.

Also we have now added a whole new appendix discussing the results.

Q2: Next, the results are not exclusively dependent on EEstd but different choice ϕ for excitatory neurons in Eq 5 can also give non zero solutions.

R2: We respectfully disagree on this point. We assume that the Editor is suggesting removing STD from EE connections and proposes a different transfer function for the inhibitory population, let us call it $\psi(x_0^I)$.

If we consider the stationary homogeneous equations (Eq. (5)) without STD by setting $w_0 \equiv 1$, and we allow for two **arbitrary** transfer functions: ϕ for excitatory neurons and ψ for inhibitory

neurons (please note that we do not assume any particular shape of the transfer functions). The stationary equations then read as

$$x_0^E = \sqrt{N} J_0 j_E \left(\sqrt{c_E}\phi(x_0^E) - g_E\sqrt{c_I}\psi(x_0^I)\right) + I_0 \tag{1}$$

$$x_0^I = \sqrt{N} J_0 j_I \left(\sqrt{c_E}\phi(x_0^E) - g_I\sqrt{c_I}\psi(x_0^I)\right) + I_0. \tag{2}$$

Since the partial excitatory and inhibitory input currents diverge as $\sqrt{N}$, a finite solution in the limit $N \to \infty$ with weak external input $I_0 = \mathcal{O}(1)$ is possible only if the terms in parentheses vanish. This yields the two conditions

$$\sqrt{c_E}\phi(x_0^E) - g_E\sqrt{c_I}\psi(x_0^I) = 0 \tag{3}$$

$$\sqrt{c_E}\phi(x_0^E) - g_I\sqrt{c_I}\psi(x_0^I) = 0. \tag{4}$$

For the generic case where $g_E \neq g_I$, subtracting these equations immediately implies

$$\psi(x_0^I) = 0 \quad \text{and hence} \quad \phi(x_0^E) = 0.$$

Therefore, in the absence of STD, the only admissible solution in the thermodynamic limit under weak external input is the trivial one with vanishing firing rates, **independently** of the specific functional forms of ϕ and ψ. Non-zero solutions exist only in the degenerate case $g_E = g_I$, which corresponds to a fine-tuned scenario. It is precisely this type of fine tuning that the nonlinear interaction introduced by STD is designed to avoid. This result demonstrates that the emergence of finite self-sustained activity under weak input is not a generic consequence of the choice of transfer functions, but instead relies crucially on the nonlinear synaptic dynamics introduced by excitatory-to-excitatory STD.

Q3: I was also surprised by the omission of some important (maybe old) papers on self-sustained activity in balanced networks. One of the better analyses of self-sustained activity in rate based models was done by Latham et al. (J. Neurophysiol. 83(2):808-827 (2000). They showed that self-sustained activity requires intrinsically active neurons in a network. Later, Vogels and Abbott (J. Neurosci. 2005) showed self-sustained activity in a network where neurons were connected with both conductance and current based synapses. Then we (Kumar et al. 2008 Neural Computation) showed self-sustained activity in a sparse network with conductance based synapses.

R3: We thank the Editor for pointing out these articles, of which we were unaware. We have found particularly interesting the article by Latham et al. (J. Neurophysiol. 83(2):808-827 (2000)) [1] and the one by Kumar et al. 2008 Neural Computation [2].

The results reported in these papers strongly resonate with our findings, in particular in [1] it is shown that in networks with current based (linear) synapses an irregular asynchronous (AI) state can emerge only in presence of a fraction of endogenously active cells, or equivalently in presence of a non-zero external current impinging a certain fraction of neurons.

In [2] this concept is made even more clear, in current based synapses, a small external input or pacemaker neurons are needed to have a self-sustained AI, this is not true for conductance based (nonlinear) synapses. For conductance based synapses, where the transfer functions are not monotonous, the AI state can persist for some period of time even in the absence of external inputs, and the period diverges with the system size.

The role of linear/nonlinear synapses is fundamental for the emergence of self-sustained AI in presence/absence of external current, and it has been recently examined in A. Sanzeni M.H. Histed, and N. Brunel, PRX (2022) where they have shown that for conductance based neurons the scaling of the synaptic coupling should be stronger than for current based synapses, namely the coupling should scale as $1/\log(K)$ instead that as $1/\sqrt(K)$.

We have stressed these points in two parts of the manuscript. First, we added the following sentence in the author summary:

Early theoretical work on self-sustained activity in large neuronal networks emphasized the role of intrinsically active neurons as a necessary ingredient to sustain low-rate firing in isolated systems [1]. In contrast, we propose....

We have also added a more comprehensive discussion in the conclusions section which now reads as:

The role played by current-based (linear) and conductance-based (nonlinear) synapses for the emergence of asynchronous irregular dynamics has been previously examined in [47]. In particular the authors have shown that while for linear synapses a small finite external current is needed to self-sustain the asynchronous irregular activity [1], this is not the case for conductance-based synapses. In this latter case the firing activity can be sustained for a finite time period even in the absence of external drive. Furthermore, the authors have shown that the life-time of this state diverges with the system size. These results show that a different type of nonlinearity in the neuronal response (the one associated to conductance-based mechanisms) can lead to findings that strongly resonate with our results. Furthermore, the investigation of conductance-based neurons has recently revealed [48] that in these models irregular dynamics can emerge dynamically, without finite tuning, whenever the synaptic strengths scale as $1/\log(K)$. Therefore to observe a self-sustained asynchronous firing activity definitely stronger synapses are required for conductance-based neurons than for current-based ones, where the synaptic strength are usually assumed to be $\mathcal{O}(1/\sqrt{K})$.

These findings together with our analysis indicate that nonlinear mechanisms can lead to completely new scenarios in the context of dynamically balanced theory, our paper presents one of these possible scenarios. However, further investigations are required to fully understand the role played by nonlinear responses on promoting self-sustained asynchronous irregular activity in excitatory-inhibitory cortical circuits.
